# A Biochemical Analysis of LINC00896 RNA in Cortex Neuronal Cells and Its Possible Connection to the Development of Autism

**DOI:** 10.1155/jna/5292315

**Published:** 2026-03-28

**Authors:** Rachel A. Hellmann Whitaker, Joshua J. Storey, Isabella Vannavong, Robert S. Mangiamele, Micalyn A. Trihus, Donovan D. Floyd, Trevor Rohder, Dylan L. Cleveland, Andrew Rizea

**Affiliations:** ^1^ Medical School, University of Minnesota, Minneapolis, Minnesota, USA, umn.edu; ^2^ School of Pharmacy, North Dakota State University, Fargo, North Dakota, USA, ndsu.edu; ^3^ Department of Chemistry, Bemidji State University, Bemidji, Minnesota, USA

**Keywords:** autism spectrum disorder, genetic risk factors, long noncoding RNA, transcriptome

## Abstract

Autism spectrum disorder is a complex neurological and developmental disorder that is characterized by altered brain structures and interconnectivity, which results in a vast array of psychosocial and physiological irregularities. This is due to the complex genetic topography of autism and to the intersectionality of genetic and environmental factors that contribute to the development of this disorder. To better understand the genetic factors that cause autism, the gene linc00896, which encodes a long noncoding intergenic RNA, was biochemically and biologically analyzed primarily through circular dichroism, liquid chromatography, and mass spectrometry. From this analysis, it was determined that LINC00896 RNA has a vast interactome and that through this interactome, LINC00896 RNA influences numerous cellular processes that contribute to the symptoms of autistic patients. Additionally, the structural analysis of LINC00896 RNA indicated stable but flexible secondary and tertiary structures that support the numerous binding interactions identified in the interactome. Through these empirical findings, the linc00896 gene was identified as being an important genetic factor that contributes to the development of autism.

## 1. Introduction

Autism spectrum disorder (ASD) is a complex neurodevelopmental disease that can be characterized by impaired socialization, defunct verbal and nonverbal communication, stemming behaviors that can result in repetitive actions, and extremely restrictive interests [1]. ASD is also known to have a strong genetic basis with possibly hundreds of genes contributing to the spectrum of psychosocial and neurodevelopmental impairments [2]. One of the genes recently identified as being a causative agent in the development of ASD was linc00896 [2]. The linc00896 gene encodes for a long intergenic noncoding RNA (lincRNA). lincRNAs and long noncoding RNAs (lncRNAs) are part of the vast transcriptome, which was identified through the ENCODE and GENCODE genome analysis projects that found that 50%–70% of the human genome is transcribed, but only 2% is translated [3–5]. LncRNAs are classified as having a length that is greater than 200 nucleotides, and can be transcribed from intergenic regions (lincRNAs) or from exons of protein‐coding genes in the sense or antisense orientations [6, 7]. LncRNAs are a very diverse group of noncoding RNA with more than 50,000 lncRNAs having been identified, which exist in one or more copies per cell [6].

The function of the massive amount of noncoding RNA products in the human genome is still being deciphered through ongoing research. Researchers are just beginning to understand the biological roles lncRNAs play in the cell and how they are influencing physiological processes in the human system. To determine these biological roles that lncRNAs play, their intracellular transport and cellular localization must be researched to better understand what processes they are involved with. Due to the immense diversity of lncRNAs, those that have been researched have been localized in the nucleus, cytoplasm, and/or the mitochondria, and some of them have the ability to be transported to different parts of the cell [6, 8–10]. To further build on the diversity of lncRNAs, they have been identified as being involved in cellular development, apoptosis, stress response, neurodegenerative disease, cancer, and cardiovascular dysfunction [9–16]. Due to the critical roles that lncRNAs play in many different biological and disease processes, further investigations into their biological functions and structures are needed to better understand the biochemical roles that lncRNAs play in the cell and in disease pathogenesis.

Herein, we are aimed at biochemically and biologically analyzing LINC00896 RNA, due to its role in ASD as well as in numerous cancers [2, 17–19]. The involvement of LINC00896 RNA in numerous diseases may be because of its ubiquitous expression profile in human tissues [20]. In the central nervous system (CNS), the linc00896 gene is preferentially expressed in the cortex and cerebellum [20]. This finding dovetails with previous research that the cortex, which controls higher processes such as learning, emotional control, and sensory functions, develops abnormally in ASD patients leading to cerebral cortex cells failing to make connections with cells in other parts of the brain [21]. Additionally, the cerebellum, which controls motor coordination as well as responses to pleasure, attention, and language, is impaired in ASD patients and develops abnormally by failing to make specific cerebro‐cerebellar connections in the brain [22]. These research findings further underscore the importance of conducting biochemical research into elucidating the structure of LINC00896 RNA, as well as its biological function. As such, this research effort focused on developing secondary and tertiary structures of LINC00896 RNA through computational modeling as well as through circular dichroism (CD) and thermal stability spectrophotometric analysis. Additionally, the biological function, cellular localization, and interactome network of LINC00896 RNA were elucidated through liquid chromatography (LC) and mass spectrometry (MS) (LC/MS). Through these experiments, 28 high‐fidelity protein‐binding partners of LINC00896 RNA were identified, which bind to LINC00896 RNA with high coverage values and reproducibility.

## 2. Materials and Methods

### 2.1. Materials

HiScribe T7 High Yield RNA Synthesis Kit E2040S (New England BioLabs, Ipswich, MA) was used to in vitro transcribe LINC00896 RNA via runoff transcription [23]. The LINC00896 RNA in vitro transcribed product was purified using the Monarch RNA Cleanup Kit T2050 (New England BioLabs, Ispwich, Massachusetts) and quantitated based on absorbance at 260 nm using a molar absorption coefficient of 23,536.54 mM^−1^ cm^−1^ [24–26]. The purified LINC00896 RNA was denatured at 80°C for 1 min, followed by an addition of 1‐mM MgCl_2_ (final concentration of MgCl_2_ was 10 mM) and quick‐cooled on ice for folding [25].

The pLINC00896 plasmid construct was created by Integrated DNA Technologies, Inc. (Coralville, Iowa). The linc00896 gene was synthesized as a 2207‐nt sequence containing a T7 RNA polymerase promoter sequence (5 ^′^‐TAATACGACTCACTATAGGG‐3 ^′^) at the 5 ^′^ end and a HindIII restriction enzyme recognition sequence (5 ^′^ ‐AAGCTT‐3 ^′^) at the 3 ^′^ end of the linc00896 gene. The synthesized linc00896 gene construct was cloned into the pUCIDT (Amp) Golden Gate plasmid, which is a standard cloning vector created by Integrated DNA Technologies, Inc. (Coralville, Iowa). The resulting plasmid is referred to as pLINC00896 throughout this publication.

Biotinylation of LINC00896 RNA was done using the Pierce RNA 3 ^′^ End Biotinylation Kit (Thermo Fisher Scientific, Inc., Waltham, Massachusetts). Each biotinylation reaction contained 50 pmol of LINC00896 RNA for a total of 1000 pmol of biotinylated LINC00896 RNA product. The biotinylation reaction protocol provided with the kit was followed exactly with a 1‐h room temperature incubation followed by an overnight incubation at 16°C. After the incubation period, a chloroform–isoamyl alcohol (24:1) extraction was done followed by an ethanol precipitation.

Primary rat cortex neurons (Thermo Fisher Scientific, Inc., Waltham, Massachusetts, Cat # A1084001) were lysed and the cell extract was used to determine the protein‐binding partners of LINC00896 RNA via affinity column chromatography.

Dynabeads MyOne Streptavidin T1 beads (Thermo Fisher Scientific, Inc., Waltham, Massachusetts, Cat # 65604D) were used to bind and immobilize the biotinylated LINC00896 RNA and then act as the stationary phase of column chromatography to capture protein‐binding partners of LINC00896 RNA from the cell lysate of primary rat cortex neurons.

### 2.2. Methods

#### 2.2.1. In Vitro Transcription Protocol

To generate a DNA template for in vitro transcription, pLINC00896 was used to transform Escherichia coli DH5a competent cells (Thermo Fisher Scientific, Inc., Waltham, Massachusetts). One resulting transformant was used to inoculate 5‐mL Luria–Bertani medium containing 100 g/mL ampicillin (LB‐Amp) and grown overnight at 37°C while shaking. A ZymoPURE Plasmid Miniprep Kit D4209 (Zymo Research, Irvine, California) was used to isolate and purify pLINC00896. To prepare pLINC00896 for runoff in vitro transcription, pLINC00896 was digested with HindIII (Promega, Madison, Wisconsin) for 3 h at 37°C with a total volume of 80 mL. Digestion of pLINC00896 by HindIII was confirmed through agarose gel electrophoresis. The resulting digested pLINC00896 was used as a template for in vitro transcription following the protocol given with the HiScribe T7 High Yield RNA Synthesis Kit E2040S (New England BioLabs, Ipswich, Massachusetts). The volume of the in vitro transcription reaction was 100 mL and was incubated at 37°C for 7 h. The in vitro transcribed LINC00896 RNA was purified following the protocol given with the Monarch RNA Cleanup Kit T2050 (New England BioLabs, Ipswich, Massachusetts) and quantitated based on absorbance at 260 nm using a molar absorption coefficient of 23,536.54 mM^−1^ cm^−1^ [24–26]. LINC00896 RNA was then denatured at 80°C for 1 min, followed by the addition of 10‐mM MgCl_2_ and then placed on ice to promote RNA folding [25].

#### 2.2.2. Structural Characterization of LINC00896 Using CD

Structural analysis of LINC00896 RNA was done using CD to determine the relative helical and stem–loop secondary structural characteristics of LINC00896 (Mg^2+^). The concentration of LINC00896 RNA was 0.5 *μ*M with a MgCl_2_ concentration of 10 *μ*M and a total volume of 1000 *μ*L [25]. A JASCO J‐815 CD spectrophotometer (Jasco, Inc., Easton, Maryland) set at 25°C using 50 nm/min scan speed, 0.5‐s time constant with a 1‐nm bandwidth was used to analyze LINC00896 (Mg^2+^) [25, 27]. CD scans were done in triplicate with averages taken of each scan and then plotted. The resulting plots were graphed using GraphPad Prism 9 (GraphPad Software, Inc., San Diego, California).

#### 2.2.3. Characterization of the Thermal Stability of LINC00896 (Mg^2+^)

LINC00896 RNA (Mg^2+^) was kept on ice prior to conducting the thermal stability assay. The LINC00896 RNA (Mg^2+^) complex was generated as previously described [25]. The LINC00896 RNA (Mg^2+^) concentrations were 0.5 mM and the concentration of MgCl_2_ was 10 mM, all of which were kept constant throughout the temperature gradient (20°C–95°C) of the thermal stability assay. The total volume of each sample was 1000 mL, with nanopure water being the solvent. A UV‐Vis Spectrophotometer (UV‐1800 Shimadzu, Kyoto, Japan) was used to take absorbance readings at 260 nm for each sample; readings were taken at 5°C increments. All absorption readings were taken in triplicate and subsequently graphed with GraphPad Prism 9 (GraphPad Software, Inc., San Diego, California).

#### 2.2.4. Biotinylation of LINC00896 RNA and Affinity Column Chromatography

Identifying the protein‐binding partners and therefore elucidating the biological function of LINC00896 RNA required biotinylating LINC00896 RNA and then binding it to Dynabeads MyOne Streptavidin T1 beads. The Dynabeads MyOne Streptavidin T1 beads with immobilized biotinylated LINC00896 RNA acted as the stationary phase of an affinity column chromatography experiment. As previously described, biotinylation of LINC00896 RNA was done using the Pierce RNA 3 ^′^ End Biotinylation Kit (Thermo Fisher Scientific, Inc., Waltham, Massachusetts). Each biotinylation reaction contained 50 pmol of LINC00896 RNA that was used to create a total of 1000 pmol of biotinylated LINC00896 RNA product. The biotinylation reaction protocol provided with the kit was followed exactly with a 1‐h room temperature incubation followed by an overnight incubation at 16°C. After the incubation period, a chloroform–isoamyl alcohol (24:1) extraction was done followed by an ethanol precipitation. Once the biotinylated LINC00896 RNA pellet was dry, 20 mL of nanopure water was added to each reaction tube.

To prepare the Dynabeads MyOne Streptavidin T1 beads, 0.5 mL (2.5 mg/mL) of beads were added to 0.5 mL of 1X Binding and Wash (B & W) Buffer (2X B & W Buffer: 10‐mM Tris‐HCl [pH 7.5], 1‐mM EDTA, 2‐M NaCl), and then centrifuged at 5000 rpm for 1 min at 4°C. We removed and discarded the supernatant, and repeating this two more times for a total of three washes. The Dynabeads MyOne Streptavidin T1 beads were then washed two times with Solution A (0.1‐M NaOH and 0.05‐M NaCl). The supernatant was removed and discarded. Then the Dynabeads MyOne Streptavidin T1 beads were washed one time with Solution B (0.1 M NaCl), and the supernatant was removed and discarded. The biotinylated RNA was added to the Dynabeads MyOne Streptavidin T1 beads along with 40 U of RNase Inhibitor. The mixture was incubated at room temperature while shaking for 1.5 h and then at 4°C overnight while shaking. The LINC00896 RNA bound Dynabeads MyOne Streptavidin T1 beads were then centrifuged at 5,000 rpm at 4°C, and then the unbound RNA was removed with the supernatant. A total volume of 0.5 mL of RNA–streptavidin interaction buffer (20‐mM Tris‐HCl [pH 7.5], 300‐mM KCl, 0.2‐mM EDTA, 0.5‐mM DTT, 0.5‐mM PMSF, and 40‐U RNase Inhibitor) was then added to the LINC00896 RNA bound Dynabeads MyOne Streptavidin T1 beads [28]. The beads were resuspended and then centrifuged at 5000 rpm for 1 min at 4°C; the wash was repeated two more times [28]. The beads were resuspended in 0.5 mL of RNA–streptavidin interaction buffer and incubated at 4°C while shaking.

The primary rat cortex neuron cells (1 mL) were centrifuged at 5000 rpm for 5 min at 4°C, the supernatant was removed, and then the cells were resuspended in 0.5‐mL mammalian lysis buffer (0.1‐M EDTA [pH 8.0], 0.5% SDS, and 10‐mM Tris‐HCl [pH 8.0]) [29]. The cells were then sonicated at 35 Amp for 1.5 min with 10‐s pulses. The lysed cells were centrifuged at 5000 rpm for 30 min at 4°C [29]. The cell lysate supernatant was then applied to the LINC00896 RNA bound Dynabeads MyOne Streptavidin T1 beads, and the mixture was incubated overnight at 4°C while shaking with 40 U of RNase Inhibitor [28].

Elution of the proteins bound to the LINC00896 RNA–steptavidin bead complex started with the centrifugation of the beads for 1 min at 5000 rpm at 4°C [28]. The supernatant was collected, and 0.5 mL of RNA–streptavidin interaction buffer was added to the beads and resuspended [28]. The resuspension was centrifuged for 1 min at 5000 rpm at 4°C, the supernatant was collected, and repeated two more times. Finally, 150 mL of 0.1% SDS was added to the pelleted beads and then were resuspended [28]. To elute the bound proteins from the LINC00896 RNA–steptavidin bead complex, the solution was boiled for 3 min followed by a centrifugation at 5000 rpm for 1 min at 4°C [28]. The protein elution was collected and subsequently subjected to protein electrophoresis using a 10% SDS‐PAGE for protein separation. The resulting protein bands were extracted from the gel and submitted for LC/MS analysis. Additionally, a control sample was submitted for LC/MS analysis, which was a blank‐bead run of the aforementioned experimental protocol. The control experiments were essential for the generation of a baseline to access nonspecific protein binding.

#### 2.2.5. MS for the Identification of the Protein‐Binding Partners of LINC00896 RNA

The protein gel fragments were first subjected to in‐gel trypsin digestion, as previously described [30, 31]. As described previously, the gel fragments were cut into 2 mm^2^ × 1 − mm^2^ rectangular pieces, then washed with 50‐mM ammonium bicarbonate (pH 7.8) and 50% (v/v) acetonitrile at room temperature for 15 min and then aspirated [30]. This aspiration and wash step were repeated one more time. Dehydration of the gel fragments with 100% (v/v) acetonitrile was conducted at room temperature for 1 min. The acetonitrile was aspirated and 50‐mM ammonium bicarbonate (pH 7.8) with 10‐mM dithiothreitol were added to the gel fragments and incubated at 56°C for 1 h. The buffer solution was then aspirated and 55‐mM iodoacetamide was added to the gel fragments and incubated at room temperature in dark conditions for 30 min [30]. This was followed by aspiration of the buffer and then 50 mM ammonium bicarbonate (pH 7.8) and 50% (v/v) acetonitrile were added, this was again followed by aspiration and repeated two more times. Once complete, 100% (v/v) acetonitrile was added to the gel fragments and incubated at room temperature for 1 min, followed by aspiration. The next step consisted of preparing the trypsin solution, which contained 5 mg/mL sequencing grade trypsin (Promega, Madison, Wisconsin) in 50‐mM ammonium bicarbonate (pH 7.8) with 5‐mM calcium chloride. The solution was then added to the gel fragments until the fragments were fully covered by the trypsin solution. The gel fragment–trypsin mixtures were then incubated on ice for 10 min, followed by an aspiration step. The buffer containing 50‐mM ammonium bicarbonate (pH 7.8) with 5‐mM calcium chloride was then used to cover the gel fragments. The gel fragment samples were then placed in a warm air incubator at 37°C for 16 h. The resulting solution from each gel fragment sample was transferred to a sterile 1.5‐mL microcentrifuge tube, and 50% (v/v) acetonitrile/0.3% (v/v) formic acid was added to each tube to promote the further extraction of digested peptides from the gel fragments [30]. The sample extracts were pooled together, and the process was repeated with 75% (v/v) acetonitrile/0.3% (v/v) formic acid [30]. The resulting pooled digested peptide fragments were frozen at −80°C and then dried in a speed vac [30]. This process was followed by the resolubilization of the peptide mixtures, and then the peptide mixtures were desalted using the StageTip technique with 3‐M Empore styrenedivinylbenzene extraction disks, as previously described [32]. The digested peptide mixtures were then dried in vacuo.

The digested peptide fragments were then analyzed and identified with LC and MS, as previously described [33].

Peptide separations were conducted using an Easy‐nLC 1000 HPLC (Thermo Fisher Scientific, Inc., Waltham, Massachusetts) [30]. Samples that were ~200 ng, were loaded onto a 30 cm × 100 − *μ* *m* internal diameter fused silica PicoTip emitter (New Objective, Woburn, Massachusetts), which was packed in‐house with ReproSil‐Pur C18‐AQ (1.9‐*μ*m particle, 120‐Å pore; Dr. Maish GmbH Ammerbuch, Germany) at a flow rate of 1 *μ*L/min with a buffer containing 0.1% (v/v) formic acid and 2% (v/v) acetonitrile [30]. Elution of the peptides were performed using a gradient of 5%–7% buffer Solution B (A: 0.1% v/v) formic acid in water and B: 0.1% (v/v) formic acid in acetonitrile for 1 min, 7%–35% Solution B for 1 h, and 35%–60% Solution B over 5 min at a flow rate of 200 *μ*L/min [30]. The column was mounted in a nanospray source directly in line with an Orbitrap Fusion mass spectrometer (Thermo Fisher Scientific, Inc., Waltham, Massachusetts) [30]. The spray voltage was set at 2.1 kV in positive mode [30]. The heated capillary was maintained at 275°C [30]. The acquisition method combined two scan events (i.e., a full scan and a parallel reaction monitoring [PRM] event) that target the doubly and triply charged precursor ions of the HVPGGGSVQIVYKPVD and VQIVYKPVD peptides without scheduling [30]. The full scan event employed a mass‐to‐charge ratio (m/z) 380–1500 mass selection, an orbitrap resolution of 120,000 (at m/z 200), a target automatic gain control (AGC) value of 200,000, and maximum fill times of 100 ms [30]. The PRM event used an orbitrap resolution of 30,000 (at m/z 200), a target AGC value of 200,000, and maximum injection times 55 ms [30]. The precursor ion generated from each targeted peptide was isolated using an isolation window of 1.6 m/z [30]. Fragmentation was performed with a higher energy collisional dissociation collision energy of 30%. MS/MS scans were collected using a scan range from 100 to 1000 m/z. PRM data were collected in centroid mode [30].

To conduct the mass spectral database search, Peaks Studio 8.5 (Bioinformatics Solutions, Inc, Waterloo, Ontario, Canada) was used for interpretation of MS/MS (mass spectra) and protein inference [30, 34]. Search parameters were Rattus Norvegicus (Taxonomy ID 10116) protein sequence database from UniProt (http://www.uniprot.org/) downloaded on December 13, 2016 concatenated with the common lab contaminant database from http://www.thegpm.org/crap/; precursor mass error tolerance: 50.0 ppm; fragment mass error tolerance: 0.1 Da; precursor mass search type: monoisotopic; no enzyme specificity; variable modifications: methionine oxidation and dioxidation, cysteine carbamidomethylation, pyroglutamic acid, and protein N‐terminal acetylation; maximum variable modifications per peptide: two; false discovery rate calculation: on; spectra merge: off; no charge state correction; and spectral filter quality: > 0.65 [30].

Interpretation of the mass spectral data relied on a baseline for peptide detection. Support for the detection of peptides from each supporting MS/MS spectrum was based on (1) a minimum of five consecutive b‐ or y‐type peptide fragment ions, (2) 1% peptide and protein false discovery rate threshold, and (3) precursor mass accuracy < 7 ppm [30].

Human and/or animal subjects were not used to conduct this research.

## 3. Results

**Figure 1:**
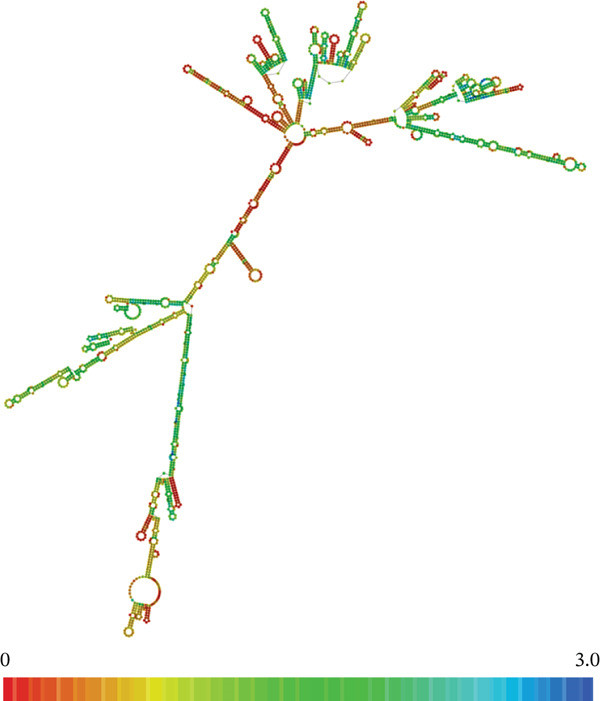
Secondary structural prediction of LINC00896 RNA. The computational secondary structural prediction of LINC00896 RNA was based on the thermodynamic ensemble prediction model available on the RNAfold WebServer platform using RNAfold 2.4.18 [39]. The free energy of the thermodynamic ensemble was −978.50 kcal/mol. The bases are color coded as determined by the positional entropy; the lowest entropy on the color gradient is red, and the highest entropy is blue [39].

The roles lincRNAs play in the cell are not well understood [35]. Therefore, it is essential that the molecular biology and biochemistry of this group of noncoding RNAs are further studied so that their roles in human development, disease pathogeneses, and many other biological processes are better understood [13, 14, 16, 36–38]. Since structure determines function, our biochemical analysis of LINC00896 RNA started with computationally developing a model of its secondary structural elements (Figure 1). Figure 1 indicates that LINC00896 RNA has a complex secondary structure, due to its large size (2207 nt). RNAfold 2.4.18 software was used to render the secondary structure of LINC00896 RNA based on positional entropy [39]. Positional entropy of each nucleotide in LINC00896 RNA was illustrated in Figure 1 through a color gradient, where red indicates low positional entropy and blue indicates high positional entropy [39]. Based on the predicted secondary structure, LINC00896 RNA has a large stable core region indicated by low‐positional entropy (red/yellow), with more dynamic regions within its peripheral secondary structures as indicated by moderate entropy (green). LINC00896 RNA has very little high entropy (blue) regions, thus the overall structure is very stable with its free energy calculated at −978.80 kcal/mol.

The tertiary structure of LINC00896 RNA was rendered using the 3dRNA/DNA modeling program (Figures 2a, 2b, and 2c) [40]. 3dRNA/DNA rendered five structures for LINC00896 RNA; of these five, the lowest energy (lowest 3dRNA score) structure was chosen [40]. The PyMol Molecular Graphics System, Version 2.0 was used to manipulate the structure of LINC00896 RNA, and POV‐ray, Version 3.7 was used to create the final rendered structures of LINC00896 RNA. The computationally predicted tertiary structure is cylindrical and compact in shape, as indicated by the structural depictions of LINC00896 RNA on the x−, y−, and z−axes. Figures 2a, 2b, and 2c are presented using a color spectrum that designates the sequence position of the nucleotides within LINC00896 RNA′s primary structure.

Figure 2Tertiary structural prediction of LINC00896 RNA. The tertiary structure was predicted using the 3dRNA/DNA modeling program[40]. (a) The LINC00896 RNA visualized along the x‐axis. (b) The LINC00896 RNA visualized along the y‐axis. (c) The LINC00896 RNA visualized along the z‐axis. All structures were manipulated with The PyMol Molecular Graphics System, Version 2.0 (Schrodinger, LLC., New York City, New York). The final rendering of the structures was done using POV‐Ray, Version 3.7 (Persistence of Vision Raytracer Pty. Ltd., Victoria, Australia). The structural color spectrum is indicative of the nucleotide position within the sequence. The Nucleotides 1–411 are blue, 411–556 are teal, 556–1176 are green, 1176–1356 are yellow, 1356–1776 are orange, and 1776–2207 are red.

**Figure:**
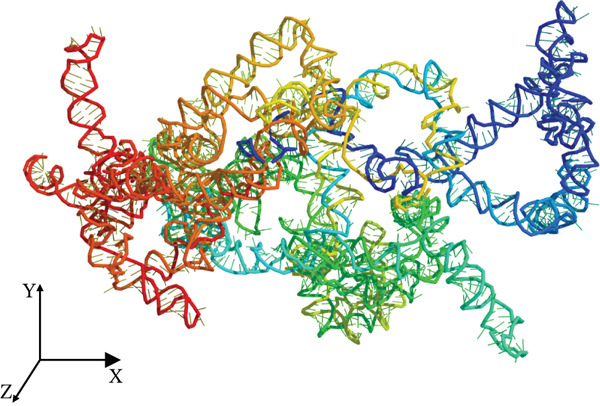
(a)

**Figure:**
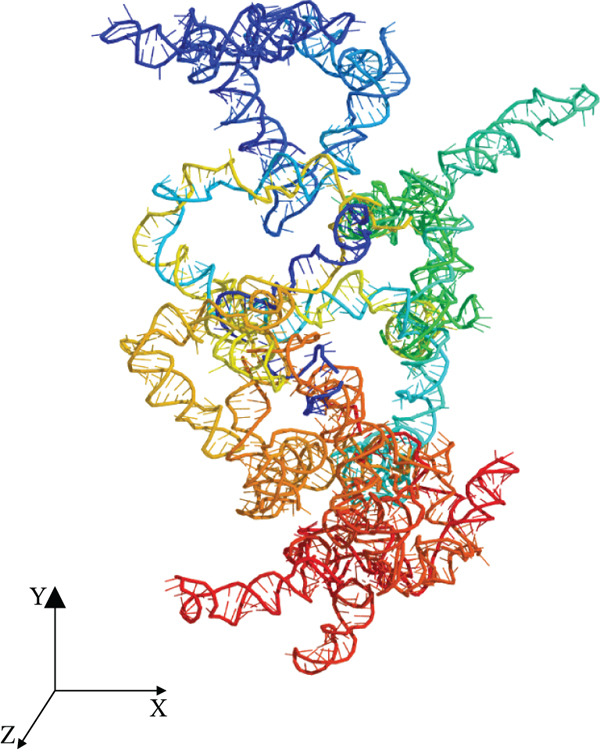
(b)

**Figure:**
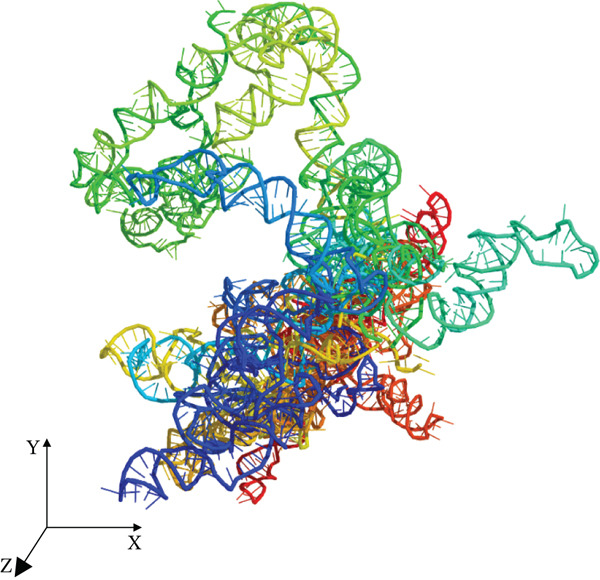
(c)

**Figure 3:**
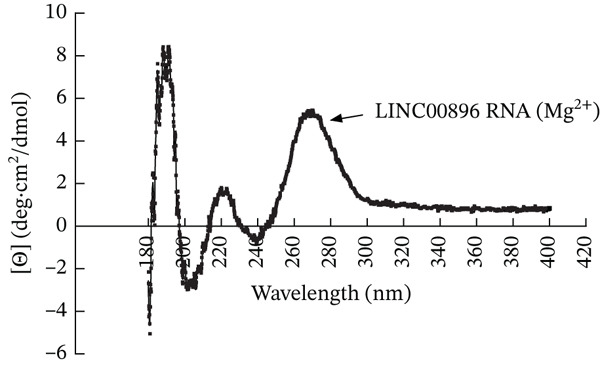
Circular dichroism spectrum of LINC00896 (Mg^2+^). Circular dichroism spectrum of LINC00896 RNA (Mg^2+^). The LINC00896 RNA concentration was 0.5 *μ*M and the MgCl_2_ concentration was 10 *μ*M in nanopure water. The LINC00896 RNA sample was scanned in triplicate using a JASCO J‐815 circular dichroism spectropolarimeter set at 25°C using 50‐nm/min scan speed, 0.5‐s time constant and a 1‐nm bandwidth [27].

Empirical structural analysis of LINC00896 RNA′s secondary structure was done using CD. Helical structures in RNA are similar to A‐form DNA helices and therefore display a positive peak near 260 nm and a negative peak near 210 nm in CD spectra [25, 41–43]. Additionally, stem–loop secondary structures are commonly formed in RNA and can be identified on CD spectra by a negative peak at around 245 nm and a positive peak in the range of 265–285 nm [43]. Furthermore, a strong positive peak near 186 nm indicates right‐handed A‐form RNA [44]. Due to the large size and complex tertiary structure of LINC00896 RNA, CD spectral analysis showed slight shifts from what would be expected (Figure 3) [45]. Shifts in CD spectra occur upon tertiary structure formation as helical secondary structural elements affect the absorption of stem–loop secondary structural elements [45, 46]. Additionally, changes in pH, ion concentration, and temperature can all cause shifts in CD spectra [45, 46].

**Figure 4:**
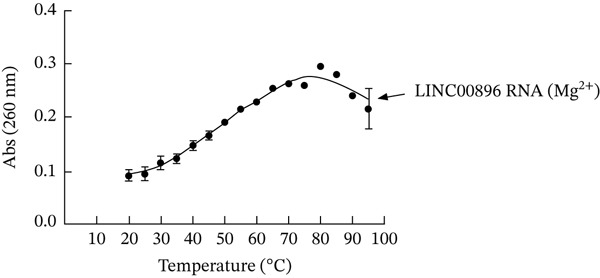
Thermal stability of LINC00896 (Mg^2+^). UV/Vis spectrum of LINC00896 RNA (Mg^2+^), measuring the denaturation of LINC00896 RNA. The LINC00896 RNA concentration was 0.5 *μ*M and the MgCl_2_ concentration was 10 mM.

Characterization of the tertiary structure of LINC00896 RNA through thermal stability studies was carried out to determine the structural stability of LINC00896 RNA (Mg^2+^). Thermal stability was plotted as a change in absorption at 260 nm versus temperature. Figure 4 indicates that LINC00896 RNA′s tertiary structure is thermally sensitive, with denaturation beginning as soon as heat stress was applied. The tertiary structure of LINC00896 RNA continues to denature until the absorption maxima is reached near 80°C, at which time the absorption values begin to decrease. The decrease in absorption values could be explained through aggregation of nucleic acid fragments as well as nucleotides that fractured off the main structure due to heat stress [47, 48]. Thermal denaturation reduces base pairing; however, the hydrophobic interactions between stacked bases can promote aggregation at high heat [47, 48].

To determine the biological function of LINC00896 RNA, affinity column chromatography and LC/MS were used. Biotinylated LINC00896 RNA was tethered to streptavidin beads while cell lysate from cortex neurons passed over the beads to immobilize the protein‐binding partners of LINC00896 RNA, which were then identified by LC/MS. Identification of the protein‐binding partners of LINC00896 RNA was essential in elucidating LINC00896 RNA′s biological function. In order to identify the legitimate protein‐binding partners of LINC00896 RNA, common protein contaminates had to be removed from the LC/MS data [49]. Additionally, a lower threshold of a 60% coverage value for identified proteins was set [50]. Figure 5a tabulates the protein‐binding partners of LINC00896 RNA that were identified with LC/MS and analyzed with Thermo Scientific Proteome Discoverer Version 3.0. The 28 proteins listed in the table were identified with high confidence among the replicates of the LC/MS experiments. Figure 5b shows that LINC00896 RNA binds numerous ribosomal proteins, as well as multiple globin, and tubulin proteins. Additionally, LINC00896 RNA binds to ATP synthase, cofilin‐1, histone, pyruvate kinase, 60‐kDa heat shock protein, phosphopyruvate hydratase, Rho GDP‐dissociation Inhibitor 1, fatty acid–binding protein, vimentin, malate dehydrogenase, ubiquitin, voltage‐dependent anion selective channel Protein 1, Glyceraldehyde 3‐phosphate dehydrogenase (GAPDH), and Rab GDP dissociation inhibitor proteins.

Figure 5Identification of the protein‐binding partners of LINC00896 RNA through LC/MS analysis. (a). Protein identification table generated by Thermo Scientific Proteome Discoverer Version 3.0 from replicated LC/MS data [51]. (b). Pie chart indicating the frequency of related protein types that are binding partners of LINC00896 RNA as indicated by Thermo Scientific Proteome Discoverer Version 3.0 analysis [51].

**Figure:**
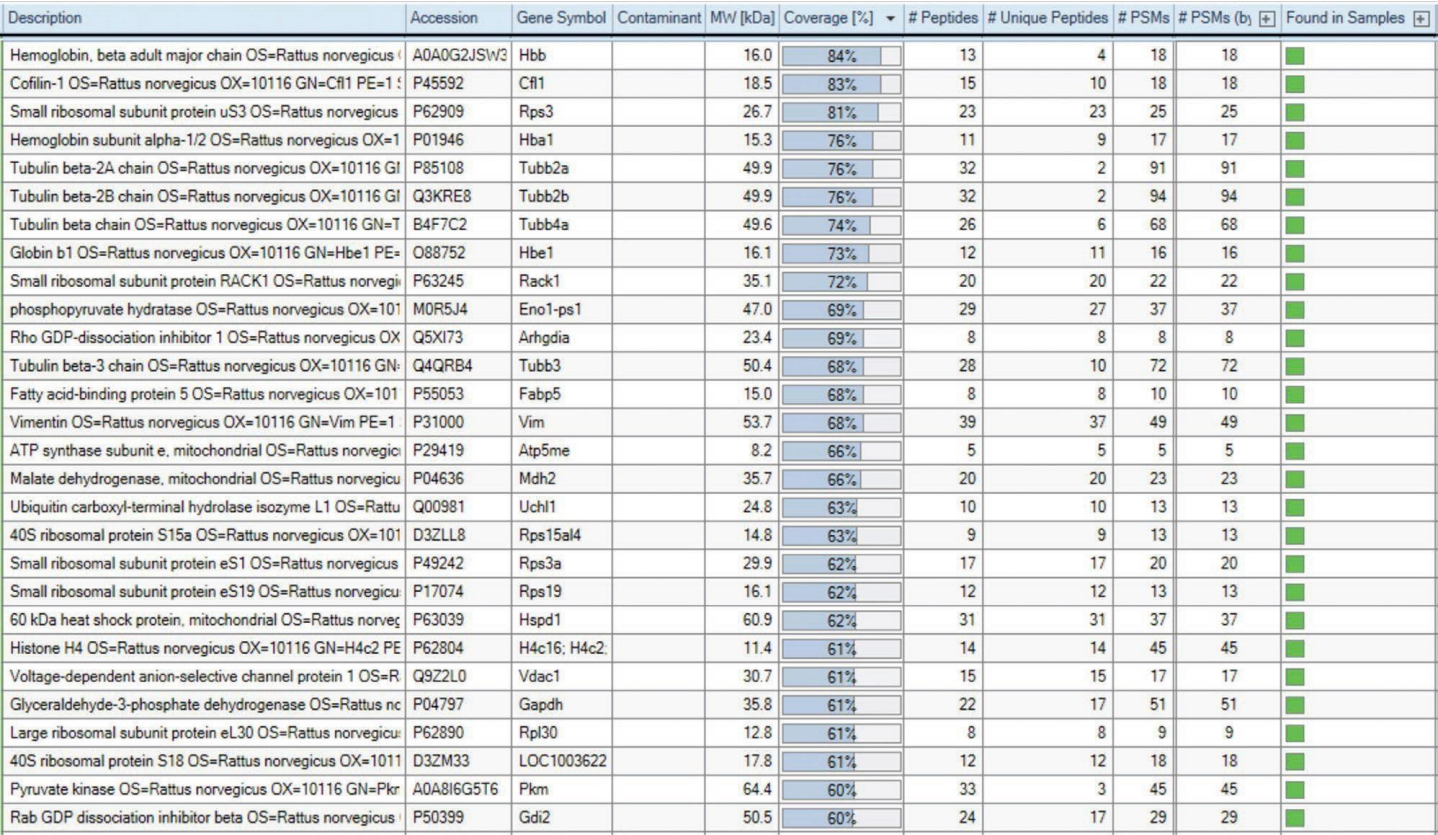
(a)

**Figure:**
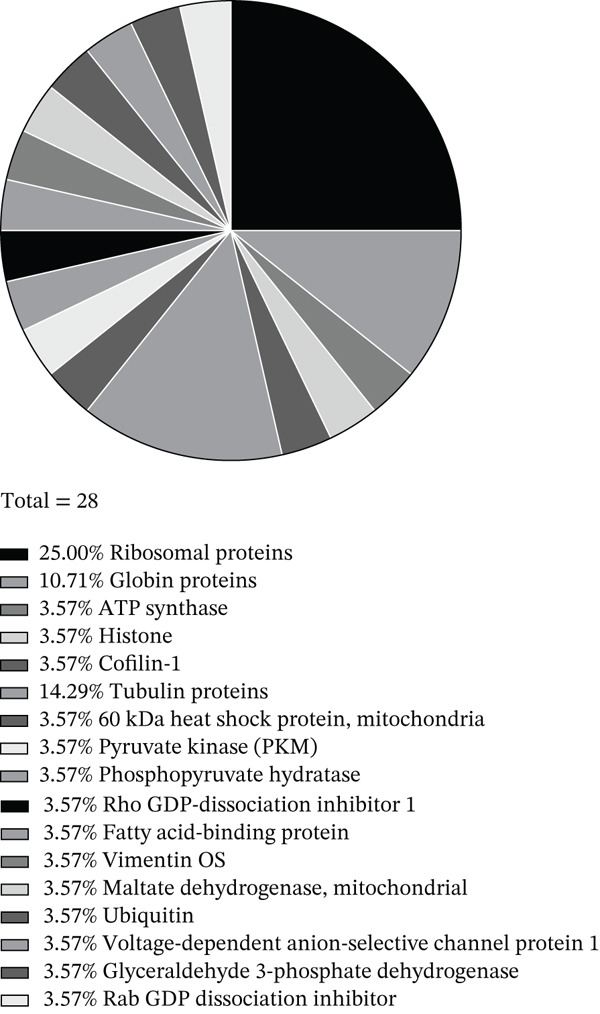
(b)

**Figure 6:**
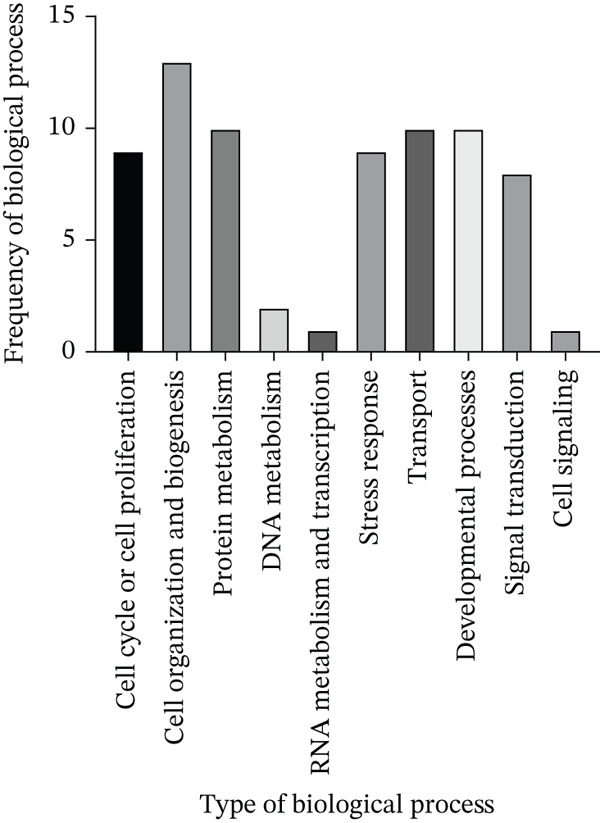
Biological processes of the protein‐binding partners of LINC00896 RNA. Data analysis by Thermo Scientific Proteome Discoverer Version 3.0 indicated that the protein‐binding partners of LINC00896 have a diverse range of biological processes in which they are involved. Data represented in this graph are based on replicated LC/MS experiments.

The next step in analyzing the protein‐binding partners of LINC00896 RNA is to determine the biological processes that are facilitated by these proteins, which will shed light on the biological function of LINC00896 RNA. LINC00896 RNA′s predominant biological function is to affect cell organization and biogenesis as indicated in Figure 6. To promote these processes, LINC00896 RNA binds to ribosomal proteins, tubulin proteins, cofilin‐1, ubiquitin carboxyl‐terminal hydrolase isozyme, 60‐kDa heat shock protein, histone H4, and GAPDH.

Protein metabolism, transport, and developmental processes are the next most predominant biological processes that are influenced by LINC00896 RNA. Protein metabolism is carried out by the ribosomal proteins, ubiquitin carboxyl‐terminal hydrolase isozyme, 60‐kDa heat shock protein, and GAPDH. Transport is facilitated by the globin proteins, cofilin‐1, fatty acid–binding Protein 5 (FABP5), ATP synthase subunit e, ubiquitin carboxyl‐terminal hydrolase isozyme, 60‐kDa heat shock protein, voltage‐dependent anion‐selective channel Protein 1, and Rab GDP dissociation inhibitor beta. Developmental processes are carried out by tubulin proteins, ribosomal proteins, cofilin‐1, hemoglobin subunit alpha, vimentin, ubiquitin carboxyl‐terminal hydrolase isozyme, and 60‐kDa heat shock protein.

LINC00896 RNA also binds to proteins that influence cell cycle or cell proliferation and stress response. The biological processes of cell cycle and cell proliferation are carried out by cofilin‐1, ubiquitin carboxyl‐terminal hydrolase isozyme, 60‐kDa heat shock protein, the ribosomal proteins, and the tubulin proteins. The stress response is facilitated by 60‐kDa heat shock protein, cofilin‐1, vimentin, ubiquitin carboxyl‐terminal hydrolase isozyme, voltage‐dependent anion‐selective channel Protein 1, GAPDH, and ribosomal proteins.

LINC00896 RNA may also influence the biological processes of signal transduction, DNA metabolism, RNA metabolism, and transcription, and cell signaling. Signal transduction is carried out by small ribosomal subunit protein RACK1, Rho GDP‐dissociation Inhibitor 1, tubulin beta‐3 chain, small ribosomal subunit protein eS19, 60‐kDa heat shock protein, GAPDH, and Rab GDP dissociation inhibitor beta. DNA metabolism is influenced by small ribosomal subunit protein uS3 and 60‐kDa heat shock protein. RNA metabolism and transcription are influenced by small ribosomal subunit protein eS19, whereas cell signaling is carried out by voltage‐dependent anion‐selective channel Protein 1.

**Figure 7:**
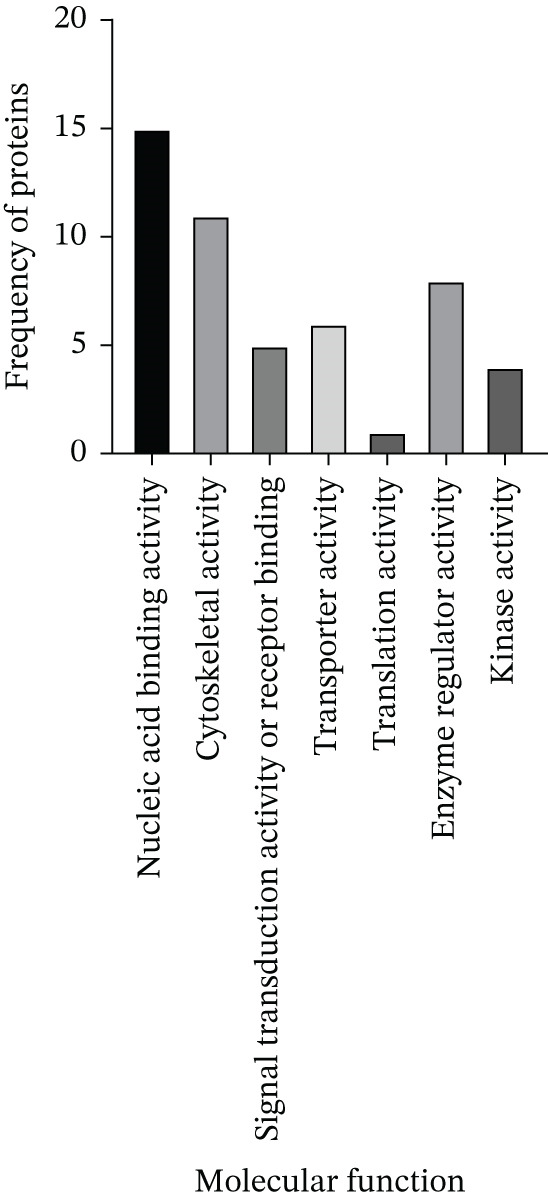
The molecular functions of the protein‐binding partners of LINC00896 RNA. Through an analysis using Thermo Scientific Proteome Discoverer Version 3.0 software, the molecular functions of each of the protein‐binding partners of LINC00896 RNA were identified. Data represented in this graph are based on replicated LC/MS experiments.

LINC00896 RNA was also found to influence the molecular functions of cortex neuronal cells, as indicated in Figure 7. Overwhelmingly, the predominant molecular function was nucleic acid–binding activity. The proteins that have nucleic acid–binding activity include histone H4, small ribosomal subunit proteins uS3, eS1, eS19, 60‐kDa heat shock protein, and the 40S ribosomal protein S18. LINC00896 RNA was also found to bind to proteins that influence cytoskeletal activity; these proteins are cofilin‐1, small ribosomal subunit protein uS3, tubulin proteins, vimentin, and glyceraldehyde‐3‐phosphate. Enzyme regulator activity was also found to be influenced by LINC00896 RNA through binding interactions with small ribosomal subunit protein RACK1, Rho GDP‐dissociation Inhibitor 1, GAPDH, and Rab GDP dissociation inhibitor beta. Additionally, LINC00896 RNA binds proteins that influence transporter activity; these include FABP5, ATP synthase subunit e, and voltage‐dependent anion‐selective channel Protein 1. Furthermore, the molecular functions of signal transduction activity or receptor binding were found to be influenced by small ribosomal subunit protein RACK1, tubulin beta‐3 chain, and ubiquitin carboxyl‐terminal hydrolase isozyme L1. Lastly, the molecular functions of translation activity and kinase activity were also identified as cellular processes impacted by LINC00896 RNA, which binds to pyruvate kinase and the small ribosomal subunit protein RACK1.

**Figure 8:**
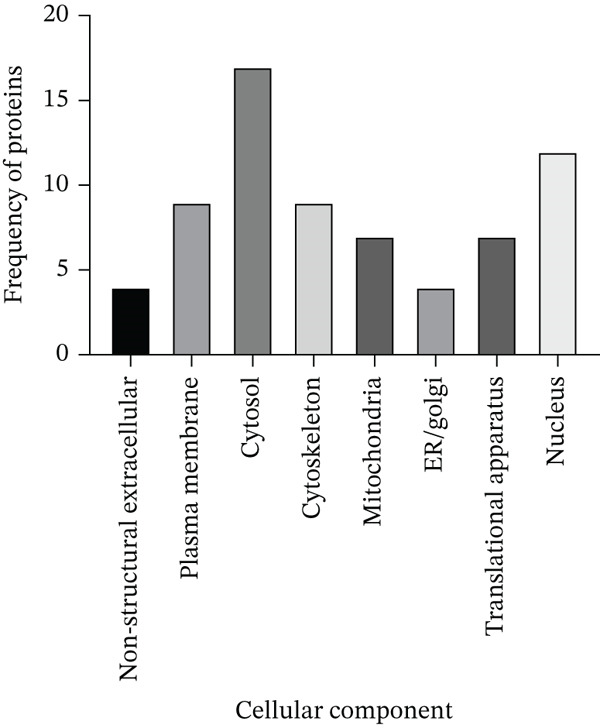
Cellular components associated with the protein‐binding partners of LINC00896 RNA. As determined by a Thermo Scientific Proteome Discoverer Version 3.0 analysis, the identified protein‐binding partners of LINC00896 RNA are associated with specific cellular components and compartments. Data represented in this graph are based on replicated LC/MS experiments.

To determine the cellular localization of LINC00896 RNA, Thermo Scientific Proteome Discoverer Version 3.0 was used to identify the cellular components associated with each of LINC00896 RNA′s protein‐binding partners. Figure 8 indicates that the protein‐binding partners of LINC00896 RNA are predominantly associated with the cytosol and the nucleus. Those that are associated with the cytosol included the globin proteins, the ribosomal proteins, phosphopyruvate hydratase, Rho GDP‐dissociation inhibitor, FABP5, vimentin, ubiquitin carboxyl‐terminal hydrolase isozyme L1, 60‐kDa heat shock protein, histone H4, and GAPDH. Those that are associated with the nucleus included cofilin‐1, the small ribosomal proteins, Rho GDP‐dissociation Inhibitor 1, FABP5, vimentin, ubiquitin carboxyl‐terminal hydrolase isozyme L1, histone H4, GAPDH, and large ribosomal subunit protein eL30. The proteins associated with plasma membrane included cofilin‐1, small ribosomal subunit proteins uS3 and RACK1, Rho GDP‐dissociation Inhibitor 1, vimentin, ubiquitin carboxyl‐terminal hydrolase isozyme L1, 60‐kDa heat shock protein, voltage‐dependent anion‐selective channel Protein 1, and GAPDH. The proteins associated with the cytoskeleton included cofilin‐1, small ribosomal subunit protein uS3, the tubulin proteins, vimentin, 60‐kDa heat shock protein, and GAPDH. The proteins associated with the mitochondria included cofilin‐1, small ribosomal subunit protein uS3 and RACK1, ATP synthase subunit e, malate dehydrogenase, 60 kDa heat shock protein, and voltage‐dependent anion‐selective channel Protein 1. The proteins associated with the translation apparatus included all the ribosomal proteins. The proteins associated with the ER/Golgi cellular components included ubiquitin carboxyl‐terminal hydrolase isozyme L1, 60‐kDa heat shock protein, voltage‐dependent anion‐selective channel Protein 1, and Rab GDP dissociation inhibitor beta. Finally, the proteins associated with the nonstructural extracellular components included Rho GDP‐dissociation Inhibitor 1, FABP5, 60‐kDa heat shock protein, and Histone H4.

**Figure 9:**
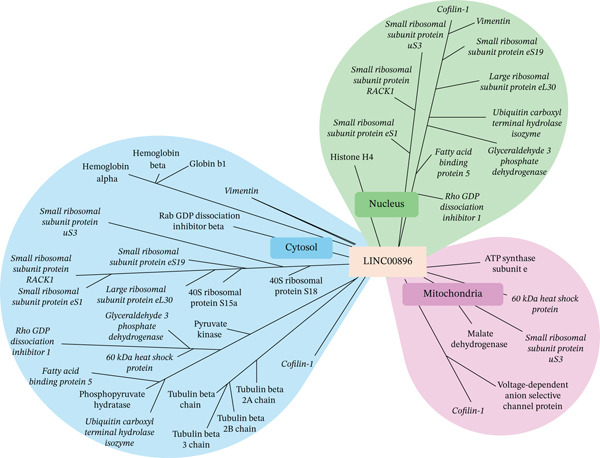
LINC00896 RNA′s interactome map. Mapping of the interactome of LINC00896 RNA was based on the cellular localizations of the protein‐binding partners of LINC00896 RNA as determined through analysis using Thermo Scientific Proteome Discoverer Version 3.0 software, as well as previous research findings. Italicized protein names are indicative of proteins that can be found in the nucleus, cytosol, and mitochondria.

After determining the cellular components that are associated with LINC00896 RNA′s protein‐binding partners, an interactome was mapped, as shown by Figure 9. The interactome was based on an analysis of the cellular components associated with each protein‐binding partner of LINC00896 RNA, as indicated by Thermo Scientific Proteome Discoverer Version 3.0, as well as through analysis of previous research on lncRNA localization [8, 10]. Through mapping the interactome of LINC00896 RNA, it was determined that it is localized within the nucleus, cytosol, and mitochondria. Likewise, numerous proteins within the interactome are also known to be localized within the nucleus, cytosol, and/or the mitochondria, as indicated by the italicized names of the proteins within the interactome map diagram.

Lastly, Figure 10 comprises LC/MS spectra of the protein‐binding partners of LINC00896 RNA that occur in high frequency with high percentage coverage, as well as a high number of peptides identified. Each of the spectra in Figure 10 is of a representative peptide from each of the high frequency protein groups that bind to LINC00896 RNA as indicated in Figure 5a,b. Figure 10a is the LC/MS spectrum of hemoglobin, beta, which had 84% coverage, and four unique peptides identified. Figure 10b is the LC/MS spectrum of cofilin‐1, which had 83% Coverage, and 10 unique peptides. Figure 10c is the LC/MS spectrum of the small ribosomal subunit protein uS3, which had 81% coverage, and 23 unique peptides. Figure 10d is the LC/MS spectrum of hemoglobin alpha, which had 76% coverage, and nine unique peptides. Figure 10e is the LC/MS spectrum of tubulin beta‐2A chain, which had 76% coverage, and two unique peptides. Figure 10f is the LC/MS spectrum of tubulin beta‐2B chain, which had 76% coverage, and two unique peptides. Figure 10g is the LC/MS spectrum of tubulin beta chain, which had 74% coverage, and six unique peptides. Figure 10h is the LC/MS spectrum of globin b1, which had 73% coverage, and 11 unique peptides. Figure 10i is the LC/MS spectrum of small ribosomal subunit protein RACK1, which had 72% coverage, and 20 unique peptides. Figure 10j is the LC/MS spectrum of phosphopyruvate hydratase (enolase), which had 69% coverage, and 27 unique peptides.

Figure 10LC/MS spectra of the protein‐binding partners of LINC00896 RNA that have the highest percentage coverage. Each spectrum is of a representative peptide from each of the 10 highest coverage proteins identified by LC/MS. (a). LC/MS spectra of the hemoglobin, beta adult major chain peptide of Residues 1–9 that was identified with high confidence with zero missed cleavage sites. (b). LC/MS spectra of the cofilin‐1 peptide consisting of the Residues 115–121 that was identified with high confidence with zero missed cleavage sites. (c). LC/MS spectra of the small ribosomal subunit protein uS3 peptide consisting of the Residues 55–62 that was identified with high confidence with zero missed cleavage sites. (d). LC/MS spectra of the hemoglobin subunit alpha peptide consisting of the Residues 18–32 that was identified with high confidence with zero missed cleavage sites. (e). LC/MS spectra of the tubulin beta‐2A chain peptide consisting of the Residues 47–58 that was identified with high confidence with zero missed cleavage sites. (f). LC/MS spectra of the tubulin beta‐2B chain peptide consisting of the Residues 47–58 that was identified with high confidence with zero missed cleavage sites. (g). LC/MS spectra of the tubulin beta chain peptide consisting of the Residues 47–62 that was identified with high confidence with zero missed cleavage sites. (h). LC/MS spectra of the globin b1 peptide consisting of the Residues 134–145 that was identified with high confidence with zero missed cleavage sites. (i). LC/MS spectra of the small ribosomal subunit protein RACK1 peptide consisting of the Residues 48–57 that was identified with high confidence with zero missed cleavage sites. (j). LC/MS spectra of the phosphopyruvate hydratase peptide consisting of the Residues 413–420 that was identified with high confidence with zero missed cleavage sites.

**Figure:**
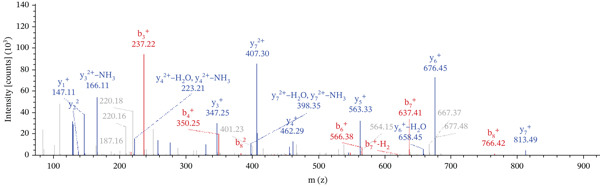
(a)

**Figure:**
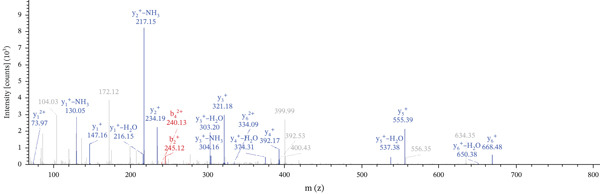
(b)

**Figure:**
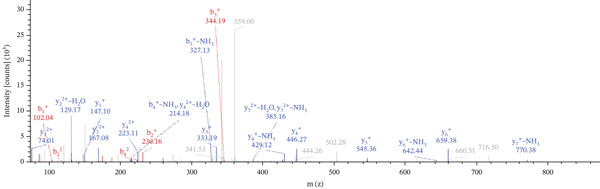
(c)

**Figure:**
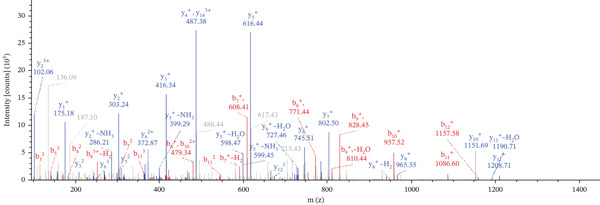
(d)

**Figure:**
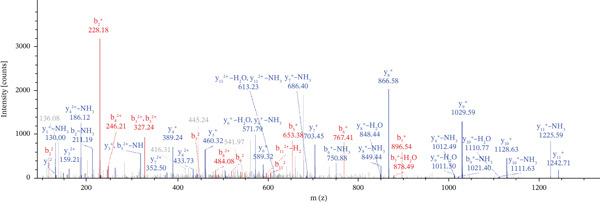
(e)

**Figure:**
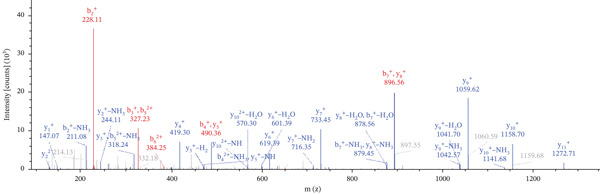
(f)

**Figure:**
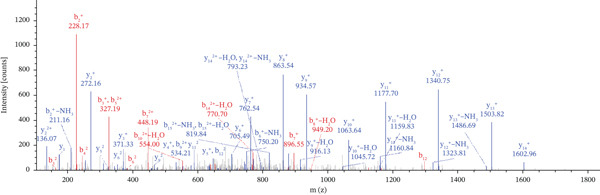
(g)

**Figure:**
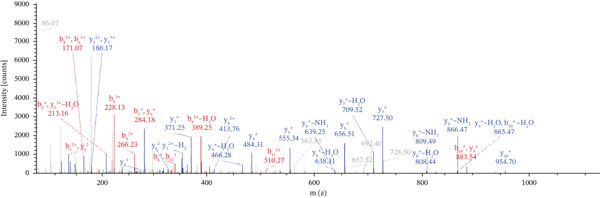
(h)

**Figure:**
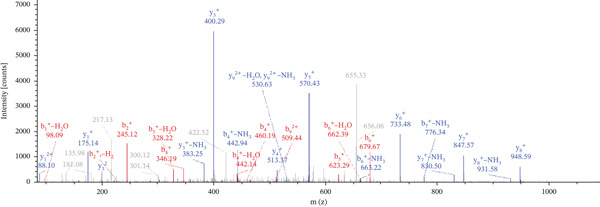
(i)

**Figure:**
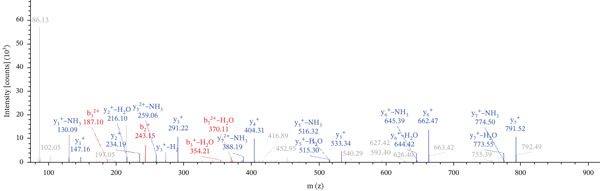
(j)

## 4. Discussion

Autism is a genetically complex developmental disorder, with only a small fraction of the causative genes being identified and thoroughly researched [2]. Further complicating the genetic landscape of ASD is that it can be driven by a multitude of genetic variants arising from across the genome that can impact a wide range of developmental functions, with individual pathologies arising from specific tissue types and cellular processes [52, 53]. To better understand the genetic landscape of ASD, the individual contributing genes must be researched for all the pieces of the autism puzzle to be put together so that genetic tests can be developed for earlier and more accurate diagnoses. The linc00896 gene was identified as being a possible causative genetic element in the development of ASD [2]. As such, the goal of this research endeavor was to better understand the structural nature and biological functions of the lncRNA, LINC00896.

The biological function of LINC00896 RNA was studied in the model system of primary rat cortex neurons. The Rattus genus does not have a sequence or structural homolog of LINC00896 RNA, which if it did, the rat homolog could have base‐paired with the human LINC00896 RNA tethered to the streptavidin beads, negatively impacting RNA–protein interactions. With this said, the highly conserved mammalian evolution that humans and rats share (approximately 88%–91% protein sequence identity) makes the rat model ideal for understanding human physiology [54]. Furthermore, the experimental results indicate that the human LINC00896 RNA only bound to rat proteins that have a direct protein (structural and sequence) homolog in the human system, which validates the experimental observations.

Structural elucidation of LINC00896 RNA was determined through computational and empirical methods. The computational secondary structural prediction of LINC00896 RNA was accomplished using RNAfold 2.4.18 [39]. Through this structural analysis it was determined that LINC00896 RNA′s structure is primarily composed of stem–loop secondary structural elements. Based on the positional entropy, the helical portions of the stem–loop secondary structural elements, which typically assume an A‐form helical confirmation, have lower positional entropy, whereas the more disordered loop confirmations have higher positional entropy, giving the overall structure a predicted free energy of −978.80 kcal/mol [45]. Thus, the secondary structure of LINC00896 RNA has numerous loop confirmations, giving rise to dynamic and flexible RNA, but the stem confirmations provide structural stability with lower positional entropy, making LINC00896 RNA an excellent binding partner for other macromolecules [45]. Typically, RNA docking sites are flexible and often are loop structures, which allow for the malleability needed to create stable interactions between the RNA and its binding partners [55, 56]. Additionally, binding partners of RNAs can cause a cascade of structural remodeling events in the targeted RNA, which when formed, rely on the structural flexibility of the RNA [45, 55]. The secondary structure of LINC00896 RNA indicates a highly stable core with the more flexible stem loop structures on the periphery, which correlates with structural regions that have greater malleability and therefore could serve as docking sites for macromolecular binding partners [45].

Further structural analysis using the 3dRNA/DNA modeling program to computationally develop a tertiary structure of LINC00896 RNA complements LINC00896 RNA′s predicted secondary structure [40, 45]. The predicted tertiary structure of LINC00896 RNA has a stable core with protruding stem loops that create flexibility. LINC00896 RNA′s overall structure is elongated with a cylindrical shape [45]. These structural features would maximize the interactions possible, which could promote docking of many different macromolecular binding partners [45].

Experimentally, structural analysis of LINC00896 RNA was done using CD and thermal stability assays [45]. CD is a technique that can be used to identify secondary structural motifs in RNA [25, 45]. Helical structures in RNA are similar to A‐form DNA helices and therefore display a positive peak near 260 nm and a negative peak near 210 nm in CD spectra [25, 41–43, 45]. Additionally, stem–loop secondary structures are commonly formed in RNA and can be identified on CD spectra by a negative peak at around 245 nm and a positive peak in the range of 265–285 nm [43, 45]. Furthermore, a strong positive peak near 186 nm indicates right‐handed A‐form RNA [44]. From Figures 1 and 2, LINC00896 RNA comprises many stem–loops, which is supported by the CD results. There were slight shifts in the CD absorption pattern due to the large size of LINC00896 RNA and its complex tertiary structure [46]. Furthermore, changes in pH, ion concentration, and temperature can all cause shifts in CD spectra [45, 46]. Even with these slight shifts, the CD spectrum indicates a large complex RNA structure that comprises many stem–loop secondary structural elements.

The thermal stability experiments support the computationally rendered structures of LINC00896 RNA, which show numerous stem–loop structural motifs that have greater flexibility and higher positional entropy [45]. These flexible regions within LINC00896 RNA contributed to its heat sensitivity and the denaturation pattern seen in Figure 4.

Therefore, the overall structural analysis of LINC00896 RNA concluded that the structure is malleable, complex, and can accommodate docking of numerous binding partners [45].

Determining the biological function of LINC00896 RNA in the context of ASD was the major focus of this research endeavor. Prior to this research, the specific biological functions of LINC00896 RNA were largely centered in the carcinogenesis of numerous types of cancer [17–19]. Further exploratory research into the genetic expression profile of LINC00896 RNA indicated that it is strongly expressed in the pituitary and thymus glands, and it is also expressed in the cerebellum and cortex (P 2014). This neurological genetic expression profile of LINC00896 RNA supports research that links LINC00896 RNA expression to the development of ASD [2]. However, the specific role that LINC00896 RNA plays in the development of ASD remains unknown. Therefore, elucidating the biological functions of LINC00896 RNA in the context of cortex neuronal cells may help to shed light on the pathogenesis of ASD as well as its role in other important disease processes.

To begin to understand the biological function of LINC00896 RNA, its protein‐binding partners needed to be identified. To this end, affinity column chromatography was carried out with LINC00896 RNA immobilized on the stationary phase, and cell lysate from primary rat cortex neuron cells comprising the mobile phase [45]. Affinity column chromatography allowed for the binding partners of LINC00896 RNA to be eluted and identified through LC/MS [45]. The high confidence, reproducible binding partners of LINC00896 RNA included 901 proteins, with the highest coverage proteins tabulated in Figure 5a. The highest coverage proteins included 28 proteins, and of this group, 25% were ribosomal proteins. Protein translation within the CNS plays a critical role in neuronal functions which include neurite growth, axon guidance, synapse formation, and synaptic plasticity [57]. Furthermore, rapid protein synthesis plays an important role in learning, memory, emotional control, neuron regeneration, and synaptic network formation [58]. In ASD, overactive protein synthesis has been observed, which is thought to lead to abnormal protein function in ASD patients; therefore, this creates a link to LINC00896 RNA′s predominant biological role in protein translation [59]. LINC00896 RNA′s role in protein translation also provides further connections with other biological processes that LINC00896 RNA may participate cell proliferation, biogenesis, protein metabolism, development, and stress responses, as indicated in Figure 6. The LC/MS results also indicated that LINC00896 RNA binds to numerous ribosomal proteins, which could explain the other related molecular functions of nucleic acid binding activity, translation activity, and enzyme regulator activity, as indicated in Figure 7.

LINC00896 RNA also binds to numerous tubulin proteins (Figure 5a,b), Tubulin proteins are known to impact the biological processes of cell organization and biogenesis, cell cycle, or cell proliferation, developmental processes, and signal transduction (Figure 6). Tubulin proteins have the molecular functions of cytoskeletal activity and signal transduction activity (Figure 7). Tubulin proteins polymerize to form microtubules, which comprise the cytoskeleton [60]. In the brain, microtubules contribute to brain development by controlling neuron production, synapse formation, and myelination [61]. In ASD patients, microtubule function is impaired, resulting in an altered brain structure that reduces myelination of oligodendrocytes as well as reduced glutamate release in synapses, thereby negatively affecting cognition, memory, and mood regulation [61, 62]. These impairments lead to some of the symptoms experienced by ASD patients [61]. Due to the binding affinity between tubulin proteins and LINC00896 RNA, these research findings may indicate a role for LINC00896 RNA in helping to regulate neuron biogenesis and organization within the brain.

Additionally, LINC00896 RNA has binding affinity for cofilin‐1 and vimentin, which are both known to play a role in cytoskeleton organization and regulation. Vimentin directly affects neuritogenesis, which is an integral step in the development of mature neuronal morphology, which may provide a link to ASD pathology since ASD patients have been found to have abnormal amounts of neurons [63, 64]. Cofilin‐1 is known to regulate cytoskeleton organization, which may be related to the pathology of ASD since cofilin‐1 is overactive in ASD patients, leading to impairments in synaptic plasticity and associative learning [65].

LINC00896 RNA may also play a role in the biological process of transport through binding to hemoglobin alpha, beta, and globin b1 (Figures 5a,b, and 6). The globin family of proteins is known to bind to oxygen and carbon dioxide gases through the help of iron‐centered heme rings, which allows them to facilitate respiration. Research has shown that ASD patients have lower levels of hemoglobin and overall iron deficiencies [66]. These interactions provide further clues that may shed light on ASD pathology. Other proteins that play a role in the biological process of transport include Rab GDP dissociation inhibitor beta, voltage‐dependent anion‐selective channel Protein 1, and ubiquitin carboxyl‐terminal hydrolase isozyme L1. Rab GDP dissociation inhibitor beta is known to regulate Rab protein trafficking between organelles, which has been proposed to be dysregulated in ASD [67, 68]. Voltage‐dependent anion‐selective channel Protein 1 is known to regulate mitochondrial metabolism through the transport of metabolites across the outer mitochondrial membrane, but research has also shown that it may also be involved in autoimmunity in ASD children [69, 70]. Ubiquitin carboxyl‐terminal hydrolase isozyme L1 is known to maintain the levels of ubiquitin monomers in brain cells and facilitates the cleavage of ubiquitinated protein remnants, a link to autism pathology remains unknown [71].

LINC00896 RNA also has binding affinity for histone H4, which has the biological function of cell organization and biogenesis and the molecular function of nucleic acid binding activity. As part of these biological and molecular functions, histone H4 is involved in the organization of DNA by forming the nucleosome subunits of chromatin. Histone H4 is also part of the histone code and is subject to chemical modifications that alter gene expression patterns. As such, research has indicated that histone H4 is linked to ASD pathology through abnormal histone deacetylation or acetylation events leading to changes in gene expression patterns in ASD [72].

LINC00896 RNA may also influence the biological processes involved in metabolism as well as the molecular processes involved in enzymatic activity through having binding affinity for phosphopyruvate hydratase (enolase), FABP5, malate dehydrogenase, GAPDH, and pyruvate kinase. Phosphopyruvate hydratase (enolase) is a glycolytic enzyme that converts 2‐phosphoglycerate to phosphoenolpyruvate. Interestingly, neuron‐specific enolase was found to be consistently elevated in ASD patients, and it has been proposed to be biomarker for diagnosis [73]. The function of FABP5 is to act as a fatty acid transporter, as well as to regulate lipid metabolism and cell growth [74]. Though FABP5 does not have a direct link to ASD, FABP4 is thought to contribute to ASD symptoms when knocked out in a mouse model system [75]. Malate dehydrogenase is known to convert malate into oxaloacetate in the citric acid cycle. In ASD patients, malate dehydrogenase may play an important role in ASD pathogenesis, as well as act as a biomarker [76]. GAPDH is a glycolytic enzyme that converts glyceraldehye‐3‐phosphate into 1,3‐bisphosphoglycerate. In fetal brains of autistic children, GAPDH is elevated and is thought to contribute to the pathophysiology of ASD, which may make it a diagnostic biomarker [77]. Pyruvate kinase is a glycolytic enzyme that converts phosphoenolpyruvate to pyruvate. Pyruvate kinase is elevated in ASD, which could be the result of lower pyruvate dehydrogenase activity, which could lead to higher concentrations of hydrogen peroxide in the mitochondria [78]. Dysfunctional mitochondria may contribute to ASD because mitochondria are integral in neuronal development [79]. Therefore, based on our research findings, LINC00896 RNA has binding affinity for numerous metabolic enzymes that play integral roles in glycolysis and the citric acid cycle, which may shed light on LINC00896 RNA′s role in metabolic processes and may provide insight into the dysfunctional metabolic processes that may contribute to the pathology of ASD.

Further analysis of LINC00896 RNA′s binding partners indicates that it may also play a role in the cellular stress response through binding to 60‐kDa heat shock protein and Rho GDP‐dissociation Inhibitor 1. The 60‐kDa heat shock protein is a mitochondrial molecular chaperone that helps other proteins fold and it regulates protein homeostasis [80]. Current research does not indicate a link between the 60‐kDa shock protein and ASD. The Rho GDP‐dissociation Inhibitor 1′s function is to regulate GTPase signaling by preventing Rho family members from dissociating from guanine diphosphate [81]. In ASD, Rho GDP‐dissociation Inhibitor 1 may impair synaptic function in the hippocampus, leading to some of the cognitive impairment symptoms in ASD patients [82].

Finally, LINC00896 RNA has binding affinity for the ATP synthase subunit e in the mitochondria. ATP synthase helps to facilitate proton transport across the inner mitochondrial membrane. In ASD, mitochondrial dysfunction has been observed leading to decreased activity of the electron transport chain and reduced expression of mitochondrial genes [83]. These research findings provide additional clues into LINC00896 RNA′s role in metabolic processes, which may provide connections to ASD pathogenesis.

Analysis of the protein‐binding partners of LINC00896 RNA has indicated that there may be a connection to ASD pathology. Additionally, through the analysis of LINC00896 RNA′s binding partners, insight was gained into the possible biological and molecular functions of LINC00896 RNA in cortex neuronal cells. Through understanding the biological functions of LINC00896 RNA′s binding partners, LINC00896 RNA may play a role in protein translation, cytoskeletal formation, transport, and metabolism, which all can impact learning, memory, emotional control, neuron regeneration, brain development, and synaptic network formation in ASD patients.

Identification of the biological and molecular functions of LINC00896 RNA′s protein‐binding partners is even more meaningful when paired with their cellular compartmentalization, which sheds light on LINC00896 RNA′s possible transport within neuronal cells [45]. As previously discussed in the Results section, we have identified the cellular components that are associated with each of the binding partners of LINC00896 RNA [45]. Due to the large number of protein‐binding partners of LINC00896 RNA, it is not surprising that there are many different cellular components associated with this group of proteins [45]. Research into the transport of lncRNAs indicates that lncRNAs can be shuttled throughout the cell [8, 84]. Due to these research findings and to the fact that there are binding proteins that are only present in specific cellular compartments, we propose that LINC00896 RNA can be transported to the nucleus and mitochondria, as well as throughout the cytoplasm. Since it has been established that LINC00896 RNA is expressed in the cerebellum and cortex of the CNS and is proposed to play a role in ASD through its involvement in brain development by binding to histone H4 (an exclusive nuclear protein), which leads to changes in gene expression patterns in ASD, it is logical that LINC00896 RNA would be found in the nucleus [2]. LINC00896 RNA may also be found in the cytosol due to its binding interactions to exclusively cytosolic proteins such as globin proteins, tubulin proteins, 40S ribosomal protein S15a and S18, Rab GDP dissociation inhibitor beta, Rho GDP dissociation Inhibitor 1, pyruvate kinase, and phosphopyruvate hydratase. Lastly, LINC00896 RNA has binding affinity for the following exclusively mitochondrial proteins: ATP synthase subunit e, malate dehydrogenase, and voltage‐dependent anion selective channel protein. Thus, LINC00896 RNA has a vast interactome that interconnects the nucleus, cytosol, and the mitochondria, allowing LINC00896 RNA to influence many cellular processes, which may provide a link to ASD pathogenesis.

In conclusion, the data presented herein provides evidence that LINC00896 RNA may play a role in the pathogenesis of ASD. LINC00896 RNA′s possible link to ASD is through its binding interactions with numerous proteins that are known to play roles in the pathology of ASD. We propose that LINC00896 RNA is shuttled throughout a cortex neuronal cell, thus allowing it to participate in protein translation, cellular transport, cytoskeletal organization, metabolism, and the cellular stress response. These cellular processes facilitate learning, memory, and emotional control through affecting neuron biogenesis and regeneration, synaptic network formation, neuronal metabolism, and myelination. Thus, LINC00896 RNA′s binding partners contribute to many of the psychosocial symptoms of ASD. More research needs to be conducted to further biochemically characterize the numerous binding interactions of LINC00896 RNA as well as to further understand its role in the pathogenesis of ASD.

## Funding

No funding was received for this manuscript.

## Disclosure

A preprint has previously been published [85].

## Conflicts of Interest

The authors declare no conflicts of interest.

## Data Availability

The data supporting the findings of this study are available within the article.
